# The double face of Morgana in tumorigenesis

**DOI:** 10.18632/oncotarget.6058

**Published:** 2015-10-09

**Authors:** Mara Brancaccio, Stefania Rocca, Laura Seclì, Elena Busso, Federica Fusella

**Affiliations:** ^1^ Department of Molecular Biotechnology and Health Sciences, University of Torino, Torino, Italy

**Keywords:** Morgana, chord containing protein, ROCK, atypical chronic myeloid leukemia, chemoresistance

## Abstract

Morgana is a chaperone protein able to bind to ROCK I and II and to inhibit their kinase activity. Rho kinases are multifunctional proteins involved in different cellular processes, including cytoskeleton organization, centrosome duplication, cell survival and proliferation. In human cancer samples Morgana appears to be either downregulated or overexpressed, and experimental evidence indicate that Morgana behaves both as an oncosuppressor and as a proto-oncogene. Our most recent findings demonstrated that if on the one hand low Morgana expression levels, by inducing ROCK II hyperactivation, cause centrosome overduplication and genomic instability, on the other hand, Morgana overexpression induces tumor cell survival and chemoresistance through the ROCK I-PTEN-AKT axis. Therefore, Morgana belongs to a new class of proteins, displaying both oncogenic and oncosuppressor features, depending on the specific cellular context.

## INTRODUCTION

**Figure 1:**
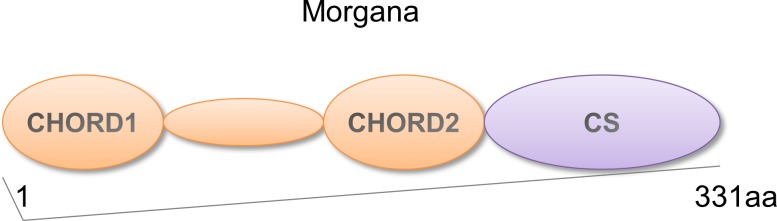
Morgana structure Schematic representation of Morgana structure. The protein is characterized by two tandemly repeated CHORD domains (CHORD1 and CHORD2) and a C-terminal CS domain.

Morgana is a protein containing two CHORD (cysteine and histidine rich) domains able to coordinate Zn^++^ ions and a C-terminal CS (after CHORD-containing proteins and *S*gt1) domain [1], homologous to the small chaperones α-crystallin and p23 (Figure 1) [2]. CHORD domains are phylogenetically conserved from plants to mammals. The first CHORD containing protein identified was the plant protein RAR1, involved in disease resistance signaling [1]. In metazoan and fungi CHORD containing proteins (Chps) acquired the CS domain, with the exception of yeast, that does not possess Chps [1]. While not vertebrates hold a single Chp coding gene, in vertebrates two genes are present, coding for Morgana and melusin [3, 4]. Mammalian Chps share the 63% homology in the amino acid sequence and a similar domain structure. Melusin expression is restricted to skeletal muscle and myocardium [3, 5], where it is involved in signal transduction leading to compensatory hypertrophy in response to increased workload [6–9]. Instead, Morgana is ubiquitously expressed and it is involved in the control of centrosome duplication and tumorigenesis [4, 10].

## MORGANA IS A CHAPERONE PROTEIN

CHORD containing proteins, RAR1 as well as Morgana and melusin, have been described to bind to HSP90 [11–18]. HSP90 is one of the best studied molecular chaperones in eukaryotes, accounting for the 2% of the total cytosolic protein content, acting as a dynamic dimer encompassing different molecular conformation shifts during its ATPase cycle. It consists of a N-terminal ATPase domain, a middle domain and a C-terminal dimerization domain. HSP90 interacts with two different classes of proteins: co-chaperones, able to assist and coordinate the HSP90 cycle and more than 200 substrate proteins, also known as clients, depending on HSP90 for their stabilization and activation. A number of co-chaperone proteins have been identified and described to associate dynamically with HSP90 during its chaperone cycle, among them SGT1, PP5, p23, prolylisomerase, Hop, Cdc37, melusin and Morgana [19]. Specific co-chaperones can inhibit or stimulate HSP90 ATPase activity, stabilize particular conformations or recruit other components of the chaperone machinery or specific subset of client proteins [19]. Many HSP90 clients are signaling proteins and transcription factors often involved in oncogenesis and tumor progression. Given that HSP90 inhibition leads to client protein degradation, HSP90 inhibitors are in clinical trials as anti-cancer treatment [20]. Morgana binds to HSP90 in its ADP bound conformation [17], suggesting it might play a role in the last phase of HSP90 cycle, eventually regulating client protein release.

Morgana, as other HSP90 co-chaperones [21], displays an HSP90 independent molecular chaperone activity in suppressing the aggregation of denatured proteins [15]. Different stress stimuli provoke unfolded proteins accumulation leading to cellular damage and death. Chaperone proteins, by inducing protein refolding, directing denatured proteins to degradation and inhibiting unfolded protein aggregation, are of crucial importance in limiting cellular damage and enhancing cell survival. Accordingly, Morgana overexpression protects cells from apoptosis induced by different stress stimuli, like heat shock and hydrogen peroxide [15]. Notably, *in vivo* experiments indicate that Morgana is upregulated, together with HSP70, in response to transient brain ischemia in gerbil hippocampus [15].

## MORGANA IN SIGNAL TRANSDUCTION AND TUMORIGENESIS

Besides HSP90, Morgana binds to Rho kinases ROCK I and ROCK II [10, 14], two proteins acting downstream of Rho GTPases [22]. Rho kinases regulate cell morphology, cell adhesion, and motility [22] by phosphorylating several downstream target proteins, including LIM kinase 1 and 2, the myosin regulatory light chain, and the myosin binding subunits of the MLC phosphatase [23]. Moreover, ROCK also regulates cell proliferation [24], differentiation [25], apoptosis [26, 27] and oncogenic transformation [23]. ROCK I and II are both ubiquitously expressed and share many downstream targets, however, differences in tissue expression levels and in substrate specificity have been reported [28]. Abnormalities in Rho kinase signaling play crucial roles in several human diseases such as cardiovascular and neurodegenerative disorders, tumor progression and metastasis [28]. ROCK activation is induced by different receptors for extracellular ligands and adhesion molecules and finely regulated by different intracellular proteins acting as activators and inhibitors. In addition to Rho, RhoGEFs and RhoGAPs, other proteins have been described to regulate ROCK, like Rnd3, Gem, C-Raf, nucleophosmin and others [28]. We demonstrated that Morgana binds to and inhibits ROCK I and II. In particular, Morgana interferes with the ability of nucleophosmin to activate ROCK II [14]. Nucleophosmin (NPM) is an ubiquitously expressed multifunctional protein, involved in a wide range of cellular processes like DNA repair, chromatin remodeling, ribosome biogenesis and centrosome duplication. NPM is often overexpressed in human solid tumors, it is involved in chromosome translocation driving hematologic neoplasms and it is the most frequently mutated gene in acute myeloid leukemia [30]. In S phase, NPM associates with and activates ROCK II, inducing centrosome duplication [31, 32]. In *morgana +/−* cells a higher amount of NPM binds to ROCK II, causing its hyperactivity and leading to centrosome overduplication [14]. Given that nucleophosmin does not bind to ROCK I [31], the precise mechanism by which Morgana inhibits this kinase needs to be clarified.

Besides Rho kinase inhibition, Morgana has been involved in the regulation of the size of dendritic arbors in Drosophila downstream of the Target of Rapamycin complex 2 (TORC2), a signaling complex comprising mTOR and Rictor, which regulates lipogenesis, glucose metabolism, actin cytoskeleton and apoptosis [33]. A further indication of Morgana involvement in metabolism and regulation of physiological process comes from the identification of Morgana transcript as a diurnal regulated gene in different brain region and in the liver [34–36].

### The importance of Morgana dosage

#### Too low

Drosophila homozygous mutants for Morgana gene (mora) die as third instar larvae due to strong defects in cell proliferation. In particular, larval neuroblasts show strong impairment in chromosome condensation and the presence of supernumerary centrosomes leading to apoptosis or genomic instability. These phenotypes are fully rescued by a human Morgana gene, indicating a conserved role of Morgana between mammals and Drosophila. Moreover, Morgana null mice die early during embryogenesis and Morgana null embryonic stem cells undergo apoptosis when blastocysts are cultured *in vitro*. Morgana heterozygous mice are viable, however primary cells obtained from these mice display centrosome amplification and genomic instability, in accordance with the phenotype of Drosophila homozygous mutants [14]. ROCK hyperactivation caused by Morgana haploinsufficiency, is responsible for this phenotype and the use of ROCK inhibitors can rescue centrosome overduplication and consequently genomic instability in *morgana +/−* primary cells [14]. This Morgana function seems to have ancient phylogenetic roots, in fact, Morgana homolog CHPA has been described to be essential for the maintenance of genome stability in Aspergillus nidulans in diploid phase [37].

**Figure 2:**
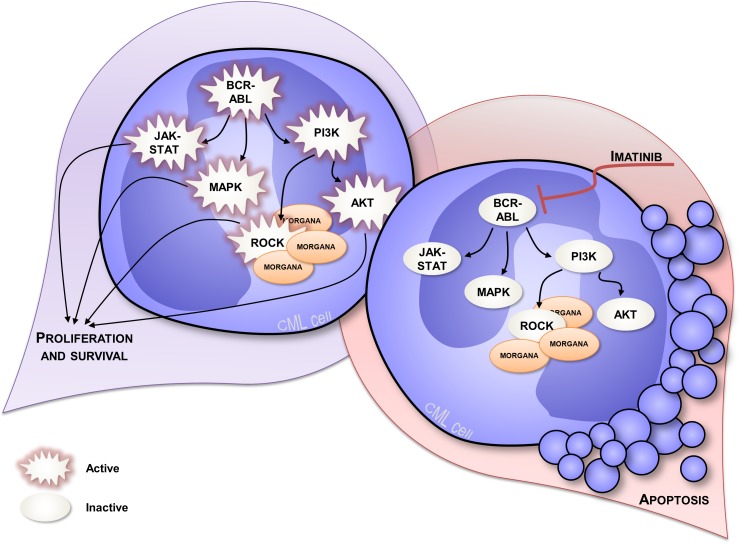
Signal transduction in CML expressing normal Morgana levels CML is caused by the presence of constitutive active kinase Bcr/Abl that leads to the hyperactivation of several signaling pathways, including PI3K, ROCK, MAPK and JAK-STAT signaling pathways [39], enhancing proliferation and survival (on the left). CML cells are addicted to Bcr/Abl signaling and Bcr/Abl inhibition using imatinib, induces apoptosis in these cells (on the right).

**Figure 3:**
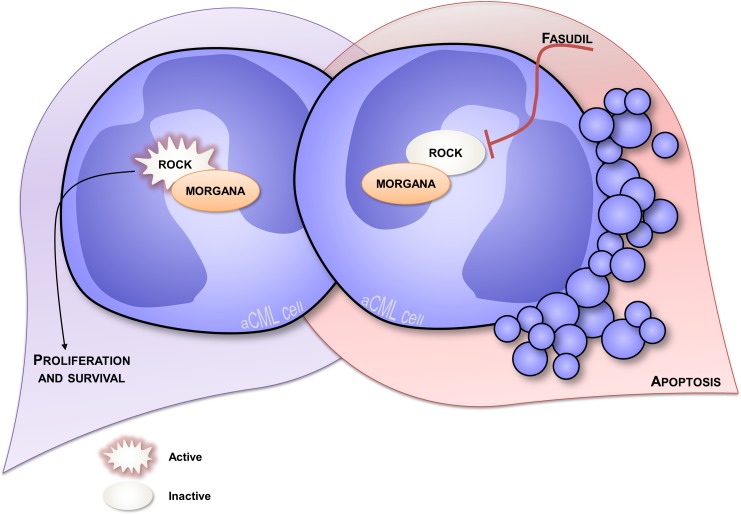
Overview of atypical CML Atypical CML bone marrow cells underexpressing Morgana are characterized by high ROCK activity which sustains survival of these cells establishing a mechanism of oncogene addiction (on the left). In fact, when ROCK is pharmacologically inhibited using fasudil, these cells can no longer survive and undergo apoptosis (on the right).

The anti-oncogenic role of Morgana was firstly disclosed by analyzing *morgana +/−* mouse embryonic fibroblasts (MEFs) that display a higher proliferation rate and oncogenic features [14]. Moreover, Morgana heterozygous mice are more susceptible to chemical induction of lung tumors and with age they develop spontaneously a lethal and transplantable myeloproliferative disease resembling human chronic myeloid leukemia (CML) [38]. In humans, CML is a myeloproliferative disorder caused, in the vast majority of cases, by the translocation t(9;22)(q34;q11) that results in the formation of the so called Philadelphia chromosome (Ph). This cytogenetic abnormality causes the fusion between BCR and ABL genes and leads to the expression of a constitutively active Bcr/Abl kinase. Imatinib, a tyrosine kinase inhibitor (TKI) able to target Bcr/Abl, is the first-line therapy in CML treatment, leading to a complete hematologic remission in the majority of patients [39] (Figure 2). However, 5% of CML patients do not present the Philadelphia chromosome and lack BCR/ABL oncogene, being affected by “atypical” CML (aCML) [40]. Of note, this disease is characterized by aneuploid karyotypes [40–42] and non recurrent cytogenetic abnormalities in the bone marrow. Morgana haploinsufficiency is able per se to drive the pathology, given that the BCR/ABL translocation does not occur in mice [43]. When we analyzed bone marrow biopsies from 5 patients affected by aCML, we found low/indetectable Morgana expression levels and high ROCK activity in all cases. In this context, reduced Morgana levels could be the causal event in inducing the pathology. The absence of specific and common targetable mutations makes aCML patients ineligible for target therapies. To date, the only therapeutic option for these patients is the treatment with conventional cytoreductive drugs with a median overall survival of 12.4 months [44]. *morgana* +/− diseased bone marrow cells are addicted to ROCK signaling and ROCK inhibitors induce apoptosis in these cells but not in wild-type controls (Figure 3) [44]. Thus, ROCK can be exploited as an innovative therapeutic target in Morgana low aCML. The fact that the ROCK inhibitor fasudil is safely used in Japan since 1995 for the treatment of cerebral vasospasm, makes this possibility even more attractive.

**Figure 4:**
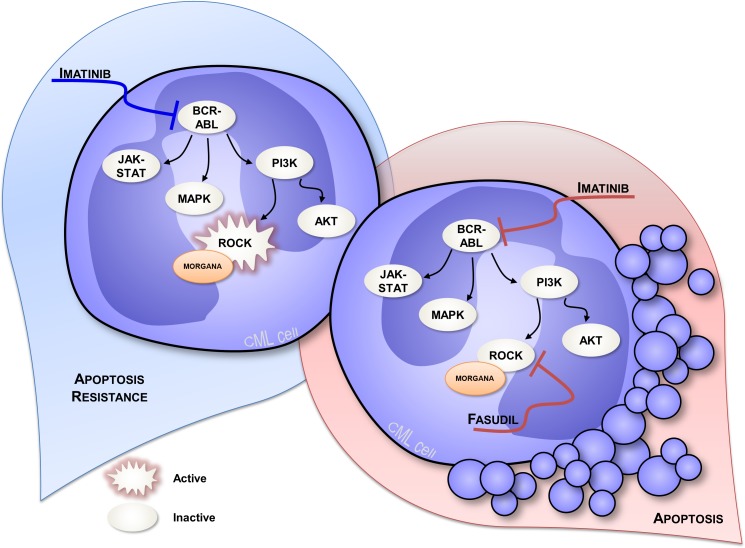
Signal transduction in CML cells expressing low Morgana levels In CML cells Bcr/Abl activates ROCK inducing addiction to its signaling and enhancing cell proliferation and survival. Morgana low expression levels cooperate with Bcr/Abl signaling to further increase ROCK activity. Consequently, low Morgana patients exhibit a sub-optimal response to imatinib (on the left). Using a combined treatment of imatinib and the ROCK inhibitor fasudil, the apoptotic response of low Morgana CML cells is restored (on the right).

Not only low Morgana expression levels can be the driving cause of human aCML, but our work highlighted that Morgana downregulation cooperates with the BCR/ABL oncogene in the 16% of CML Ph positive (Ph+) patients. Bcr/Abl is able to activate ROCK per se, and Bcr/Abl expressing cells are addicted to both Bcr/Abl and ROCK signaling [29, 45]. Since Morgana acts as a ROCK inhibitor, low Morgana levels in Bcr/Abl cells further increase ROCK activity and sustain ROCK activation also when Bcr/Abl kinase activity is inhibited by imatinib treatment, impairing apoptotic response to imatinib (Figure 4 left). Even if the majority of the Ph+ CML patients show a good response to imatinib at three months of treatment, a portion of patients exhibits a suboptimal response that has been found to be predictive of worse overall survival [46]. Our follow-up analysis indicated that low Morgana patients display a worse response during the first 24 months of imatinib treatment [38]. In this context, low Morgana levels can be used as a prognostic marker to predict a suboptimal response to imatinib treatment and to direct therapeutic intervention towards more potent, second-generation TKI like dasatinib and nilotinib. The fact that combined treatment with imatinib and ROCK inhibitors restores the apoptotic response in bone marrow cells from Morgana underexpressing patients [38] (Figure 4 right) provides the rationale for a potential use of ROCK inhibitors to enhance the response to TKI. Indeed, ROCK hyperactivation has been recently identified as a frequent signaling alteration in acute and chronic myeloid leukemia and ROCK inhibitors have been proposed as new antileukemic drugs [29, 47]. In this context, also considering the functional relationship between Morgana and NPM, it would be of interest to evaluate Morgana expression levels in acute myeloid leukemia and its role, in cooperation with oncogenes, in activating ROCK.

#### Too high

Data from lung and breast tumor tissue arrays demonstrate that besides expressing low Morgana levels, a minority of cancer samples displays Morgana protein overexpression [14]. Elevated Morgana levels in breast cancer correlate with higher tumor grade, mitosis number and lymph node involvement [10], all prognostic markers of metastatic progression [48]. Morgana overexpression is found in 16% of breast cancers without being restricted to a particular subtype.

**Figure 5:**
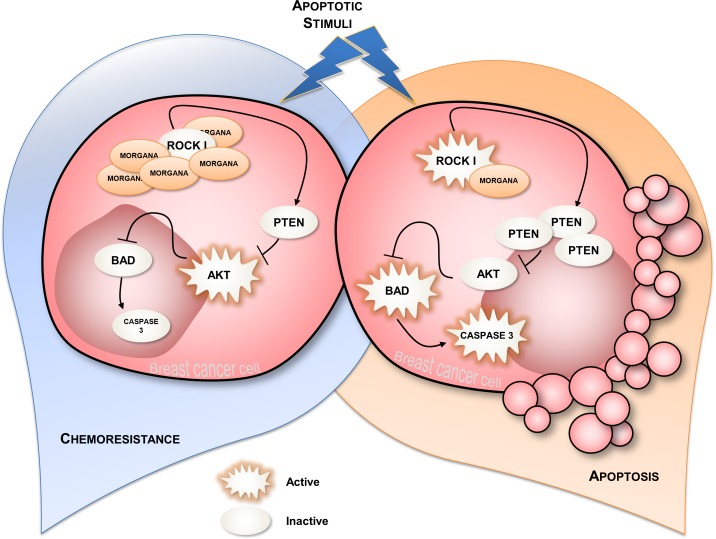
Schematic representation of Morgana normal and Morgana overexpressing breast cancer cells When breast cancer cells expressing normal Morgana levels are subjected to an apoptotic stimulus, they undergo apoptosis (on the right). Morgana overexpression, inhibiting ROCK activity, causes decreased PTEN stability and, in turn, increased AKT phosphorylation, responsible for cancer cells survival and chemoresistance (on the left).

At the cellular level, Morgana overexpression transforms NIH3T3 mouse fibroblasts and increases MCF7 breast cancer cells oncogenic properties. In particular, high Morgana levels enhance the ability of cells to withstand diverse apoptotic stimuli such as serum withdrawal, anoikis and treatment with chemotherapic drugs. Resistance to apoptosis depends on Morgana ability to inhibit ROCK I kinase activity. In particular, ROCK I can phosphorylate the oncosuppressor PTEN on serine 229 and threonine 321 [49], thereby stabilizing the protein. Accordingly, Morgana overexpression in breast cancer cells and fibroblasts induces decreased ROCK activity, PTEN destabilization and higher P-AKT levels, leading to apoptosis resistance (Figure 5). This is confirmed by the fact that downregulation of Morgana in aggressive breast cancer cells causes sensitization to docetaxel and epirubicin, two drugs used for neo-adjuvant chemotherapy. Neo-adjuvant chemotherapy is often used in breast cancer patients to improve outcomes, reduce risk of recurrence, increase tumor resectability and overall survival. However, neo-adjuvant chemotherapy treatment does also carry high risks of toxicity and is ineffective in a relevant number of patients [50]. In this context, Morgana level can be exploited as a predictive marker to direct patients toward treatment or surgery. Of note, Morgana is more frequently overexpressed in triple negative breast cancers (TNBCs) (36%) than in other breast cancer subtypes (16%) [10, 51]. TNBCs are characterized by high aggressiveness, higher rates of relapse, unresponsiveness to treatment and shorter overall survival in the metastatic setting [52]. TNBCs lack the expression of estrogen receptor (ER), progesterone receptor (PR) and the amplification of HER2 [53], for this reason, TNBC patients are not eligible for targeted therapies and adjuvant chemotherapy is the mainstay of systemic medical treatment. The fact that high Morgana levels induce AKT hyperactivation, suggests that PI3K or AKT inhibitors, already in clinical trial [54], may have a therapeutic effect in Morgana overexpressing cancers. Besides breast cancers, recent studies indicate that Morgana overexpression may play a role in the recurrence, resistance and metastatization of other cancer types (Table 1), suggesting Morgana as a general biomarker of resistance able to direct personalized therapy.

**Table 1:** Recent studies involving Morgana in cancer progression

Tumor type	Hallmark described	Reference
Breast	High Morgana protein levels confer resistance from apoptosis through ROCK-PTEN pathway	[10]
Breast	CGH analysis reveals that Morgana is frequently gained in TNBCs	[51]
Ovarian	Morgana transcript is found upregulated in recurrent ovarian cancer samples compared to primary tumors	[75]
Melanoma	Morgana transcript is overexpressed in metastatic melanoma cell lines resistant to tumor necrosis factor-related apoptosis-inducing ligand (TRAIL) compared to TRAIL-sensitive melanoma cell lines	[76]
Melanoma	Morgana transcript is associated with metastatic dissemination of cutaneous melanomas	[77]
Colorectal	Morgana transcript is upregulated in liver metastasis compared to primary colorectal cancer	[78]

## CONCLUSIONS

Deletions and amplifications in cancer cells are known to alter oncosuppressor and oncogene expression levels and lead to cancer onset and progression. Even small variations in the expression of key regulatory genes impact very relevantly on tumorigenesis [55]. However, the fact that the same gene product can cause cancer either if overexpressed (three times compared to the normal level) or underexpressed (half of the normal level), is still puzzling. This apparent paradox can be explained by keeping in mind that most proteins play multiple roles in cells. Abnormal expression of a multifunctional protein involved in both oncogenic and oncosuppressive signaling pathways can eventually lead to the prevalence of one function over the other, in a context-dependent manner. Indeed, elevated ROCK expression and activity has been detected in different types of hematopoietic [29, 47, 56, 57] and solid tumors [22, 58] and ROCK I and ROCK II activating somatic mutations have been found in cancers [59–62]. On the other hand, some studies report alteration in the expression of ROCK inhibitors and activators, like RhoE [63, 64] and RhoA [65–67], suggesting a role for ROCK inactivation in tumorigenesis.

**Figure 6:**
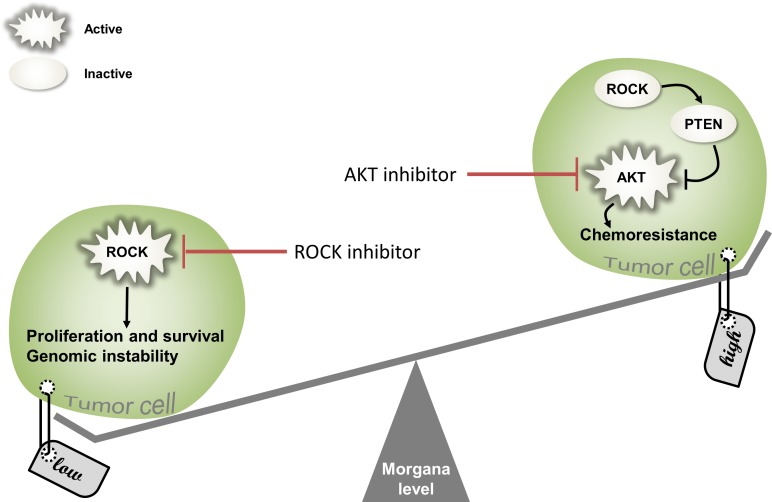
The importance of balancing Morgana levels In tumor cells Morgana low expression levels enhance ROCK activity, inducing proliferation, survival and genomic instability. ROCK inhibitors may be used as a new therapeutic approach able to rescue low Morgana levels in tumor cells (on the left). On the other hand, Morgana overexpression reduces ROCK activity and PTEN stability, enhancing AKT activation. As a consequences, Morgana high expressing cells are resistant to chemotherapy. In this context, AKT inhibitors can represent a promising approach to restore chemosensitivity (on the right).

Morgana is thus only one example of an increasingly growing class of proteins, acting both as proto-oncogenes and oncosuppressors (Figure 6), depending on their expression levels and the specific cellular context. NPM [68, 69], Wilms' tumor 1 (WT1) [70], MDM2 [71], Notch [72], Met, NF-KB, β-catenin [73], SRPK1 [74], among others, have also been described to play opposite role in tumorigenesis. In conclusion, the definition of oncogene or oncosuppressor cannot be attributed to a particular gene product, but to its specific behavior in a defined cellular context.
